# The Nursing Supplement

**Published:** 1888-08-04

**Authors:** 


### The Nursing Mirror.

All Contributions for this Supplement should be addressed "The Nursing Mirror," care of The Editor, 38, Old Jewry, E.C.

### En Passant.

A LADY DENTIST.—A well-known female dentist, who practises at the West-end, began her career as a nurse in the Franco-Prussian war. When that was over she went to America to learn dentistry, and, laden with certificates, came over to London, and was the first established lady dentist in this country. This lady is a German, though she speaks excellent English, and her habits are thoroughly American. No one could be more independent in garb or manner, and with a cigarette in her mouth she might well pass for one of the stronger sex.

ST. JOHN'S SISTERHOODS.—A correspondent points out to us that our remarks about two of the St. John's sisters who went to Canada give a mistaken impression, because of the split which once occurred in that community. To speak of the sisterhood of St. John the Evangelist and the sisterhood of St. John the Divine takes so long that irreverent outsiders have been heard to call the nurses "Brown Johns" and "Black Johns," according to the colour of their uniform. We are aware that the St. John the Evangelist sisters still carry on their good work at Charing Cross, and quite agree with our correspondent that it would be a bad day for that hospital should they ever desert it. If "Doris" had written her letter on one side of the paper only, we would gladly have published it complete; as it is, we can only explain to any of our other readers who may have been misled by us, that it was two sisters of St. John the Divine who left Charing Cross and tried Canada, and did not enjoy it.

POULTICES.—On taking up private nursing, many a hospital nurse has been surprised to learn that the ordinary method of poultice-making does not please all doctors. For instance, Dr. Lauder Brunton always instructs his nurses who have charge of medical cases to make linseed-meal poultices in the following way: Make a flannel bag twelve inches long by eight wide, open at one end with a flap to turn over. Fix a tape at each of the four corners. Mix your linseed very moistly, pour it into the bag, sew down the flap, wrap it in another piece of flannel, apply, and keep in position by tapes. Other doctors have a fancy for poultices clad in muslin, and some stick fast to the familiar tow all through their private practice. But at least it is wise for a nurse, when she has charge of a private case and is ordered to apply a poultice, to ask what method the doctor favours. Many nurses think it beneath them to inquire about such a simple thing as a poultice, and hence often have the annoyance of having their handiwork severely criticised by the doctor.

THE QUESTION OF UNIFORM.—Speaking of sisterhoods, there was an article in last Saturday's *Church Review* on "How to Reach the Masses," which pointed out the enormous influence of the Sisters of Mercy who tend temporally and comfort spiritually the sick and suffering. It stated that, clothed in their protecting garb, they could walk fearlessly amongst the most vicious; therefore, when nurses would complain of an outdoor uniform, they had better first consider whether they are throwing away an aid to good works. A nurse has opportunities of inculcating moral precepts which seldom falls to a woman's share; and, without for a moment forgetting that she is firstly a nurse, she can often give advice and help on higher matters. Hospitals are generally situated in crowded districts, and draw their patients from close at hand; if then a former patient sees his nurse going by in ordinary garb, he will fear to speak to her; they will no longer occupy th same position with regard to one anot her. But if she is still "nurse" in a cloak and veil, he may very likely come forward and seek her aid or sympathy. So long as she is at work and only off duty for a daily walk, a nurse is far better in a uniform; but if she is going away for her yearly holiday, then may the distinctive garb be well discarded. A holiday should always be a change, and a nurse should especially try to leave all memory of the sad scenes in which she usually moves behind her. And to accomplish this she should throw aside her uniform and all things likely to keep her mind in the old groove. Of course this is not possible for those who have entered the "cloistered cell," but most of our nurses are delivered from what Dr. Arnold calls "the snare and sin of perpetual vows."

THE MANCHESTER INFIRMARY.—At the annual meeting of the trustees of this infirmary, held on July 26, it was announced that an important branch of their work was that of private nursing. Their daily average of private nurses out had been a trifle over 22, and their daily averagel of private nurses waiting had been under one—in fact, to be quite exact, it had been ·50, or half a nurse. The total number of patients nursed had been 275, and of these 59 were infectious cases. The private nurses had had, with very few exceptions, excellent reports, while the nurses in the wards had also been very satisfactory. The following account of a visit to the Manchester Infirmary appeared in a Leicester paper some time ago:—"I spent one afternoon in the Manchester Royal Infirmary, and greatly pleased I was with all I saw there. The recently appointed matron was sister in one of the largest wards of our dear old hospital of St. Bartholomew. I knew her then very well, and I rejoiced to hear that Manchester had secured so suitable and excellent a matron for its important infirmary. Her sweet, gentle face retains all its freshness, notwithstanding the sootiness of her present atmosphere; her abundant hair is perhaps of a more decided grey than when last I saw her, but she is perfectly happy in her work, is well and generously supported by her committee in every effort at improvement, and is treated with much respect by her medical staff. Then her own apartments are charming, just what they should be for a lady whose life is given to looking after the welfare of others, and she took me with great pleasure and pride to see the Nurses' Home, which is in her wing of the building, quite by itself. There are about 72 nurses engaged in the infirmary nursing, and each one had her own comfortable little bedroom, where her hours of rest can be spent healthfully and alone. Excellent baths are arranged in every corridor, and in order to secure perfect quiet and undisturbed sleep to the staff of night nurses, their bedrooms are placed on the ground floor, quite isolated from the rest, and shut off from all noise during the daytime, when they are supposed to be sleeping. I was delighted with this home, and wish most heartily that such a boon could be secured for all our London hospitals, in most of which, even the best and richest, such perfect and complete accommodation is unknown. I believe the great difficulty of obtaining ground to build upon is one reason of the deficiency in St. Bartholomew's, Guy's, and other hospitals; yet I feel almost ashamed to see how very much better the Manchester nurses are cared for than are those in London. Surely it is the duty of all governing bodies to secure the health, comfort, and wellbeing of the human instruments who carry on their work, and who devote themselves to it, in spite of innumerable and preventible drawbacks on their health and strength.

### Original Articles.

### THE PROGRESS OF NURSING IN NEW YORK.

So far back as the close of the seventeenth century, Dr. Valentine Seamen used to lecture to a class of 24 nurses of the New York Hospital. From a synopsis of these lectures published in 1800, we learn that they dealt with anatomy, physiology, midwifery, &c., and that a manikin was employed in demonstration. Nevertheless the state of nursing during the beginning of this century seems to have been very bad in New York. In 1847-48 an epidemic of typhus fever devastated the city, and 700 cases were treated in Bellevue Hospital alone. Dr. Metcalfe, writing of this time, says: "Well do I remember making one morning visit, after an unusually cold night. The fever ward was warmed (?) by a stove, the fire in which had been allowed to go out. Many window-panes were lacking, a large hole was in the roof, a good-sized dog could have crawled under the outer door, and six or seven patients lay dead, whose lives in the Bellevue of to-day would have almost surely been saved. One effect of severe cold is to impair the intellectual working of the human brain. It had so benumbed that organ in our ward-tenders that they could neither find the way to the stove nor to the patients' mouths. The stimulants for the latter had unaccountably been imbibed by their guardians, nearly all of whom lay about in that happy condition in which neither consciousness of cruelty, neglected duty, nor cold, seemed to give them the least concern." This same doctor had 150 cases of typhus under his sole care at one time; and as nursing had to be done by some one, he not unfrequently spent hours in lifting, feeding, and caring for the helpless people he was trying to cure. Naturally, when the epidemic subsided there was an effort made to start a better system of nursing, and a year later we read in the hospital report:—

"Another improvement and one of incalculable importance for sound medical and moral reasons has recently been introduced here, and is already yielding the most salutary results. No longer are the sick poor condemned to the cruel infliction of being nursed by the convicts of the Female Penitentiary, nor is the public property any longer wasted and destroyed by abandoned and dissolute women whose profanity, drunkenness, and theft, have always been notorious and incurable. Hired nurses, selected from among poor females of reputable character and decent habits, are now found in all the wards of the hospital."

In 1853, St. Luke's Hospital was founded, and from its very commencement was nursed by the Protestant Episcopal Order of the Holy Communion. Its members were of a far better station than the poor "females of reputable character" who reigned at Bellevue, and of course they did far better work. Such an example was bound to be followed, and in 1873 three training schools were almost simultaneously started at New York, New Haven, and Boston. The New York School was planned by the Bellevue Hospital Visiting Committee, and a subscription fund of twenty-five thousand dollars was raised for it in the course of six weeks. The school opened with but five pupils. Applications for admission came slowly, or rather the applicants were many, but few amongst them were willing to give up two years to the acquisition of a profession, no matter how useful and lucrative that profession might prove. This, one of the most serious of the early difficulties, no longer exists; as the story of the school gradually spread through the country the applications from women deliberately choosing the profession of a nurse as their life-work, and anxious to learn it thoroughly, were more numerous than could be accepted.

In 1875 a training school for nurses was established by the Board of Public Charities and Correction, in connexion with the Charity Hospital. Upon the authority of the chief of the staff, under whose direction the school still prospers, it is stated that the death-rate of the hospital has decreased sixty per cent. in the past decade, which he attributes chiefly to the excellent nursing now given to these pauper patients. The New York Hospital, which had started so well in its youth, was at this time behind the other hospitals as regarded nursing, which was partly owing to its limited accommodation. In 1877 some new buildings were added, which included a training school for thirty probationers, and since then the school has gradually increased, and earned high commendation from physicians and the public.

Sister Helen, of University College Hospital, was the first lady superintendent of Bellevue, and she formed the training school on lines familiar to us in England, and its rules and regulations have never been greatly altered. In 1876 a nurse who had graduated at Bellevue, decided to devote herself to nursing the sick poor in their own homes, under the auspices of the City Mission. From this effort sprung the band of a dozen missionary nurses, who now perform the necessary district work in New York. They were in their early days helped by a lady from "Guy's," who had had experience of district nursing in London, but who was persuaded by the Episcopal Board of Missions to remove her talents where they were more badly needed, namely, to a hospital in China. The nurses at Bellevue are indebted to Mrs. Osborn, a charitable lady in New York, for the pleasant home, quite apart from the hospital, in which they live. This is a brick building, four storeys high, containing separate bed-rooms for sixty nurses, a dining-hall, sitting-room, and well-furnished library. The poor nurses at the Charity Hospital are obliged to live in the hospital building, and illness is constantly rife amongst them. In 1882 two of them contracted typhus fever. They have splendid prizes given for those who excel in the examinations, and they have a library of 500 volumes; but all this does not make up for the home apart from the hospital, which every training school should possess. The Mount Sinai Training School, which was opened in 1881, secured two neighbouring houses for their nurses to occupy, and started with excellent rules, except one—they take women from twenty years of age, and give them six weeks' obstetric nursing in a special institution. There is, however, a great demand for their nurses for private work, and often half the applications received have to be refused.

In 1883 the managers were forced to withdraw nurses from two wards in Bellevue, in order to employ them in the more remunerative work of private nursing. This drew from the Medical Board the following expression of appreciation: "Resolved, that in view of the great benefit conferred upon this hospital by the Training School, in view of the loss sustained by the surgical cases in the withdrawal of the trained nurses from wards 1 and 2, in view of the services rendered the general public by trained nurses who go out in the city to nurse the sick of all classes, it is the feeling of this Board that the managers of the Training School are justified in making an earnest appeal to the public for aid.

"Moved, that the resolution be signed by the President and Secretary, and a copy transmitted to the Training School.

"A. Clark, *President.*

"Fredk. R. S. Drake, *Secretary, pro tem.*"

This period of depression is now over, and the school is in a thoroughly flourishing condition, with a list of nearly 300 graduates on its books.

Five years ago a College of Midwifery was duly incorporated in New York, and opened its first session with eleven students. Instruction is given by lectures and demonstrations for three months, and two months are then spent in practical work in the Maternity Hospital. After a thorough examination in anatomy, physiology, midwifery and puerperal diseases, and the care of infants, a signed diploma is given to successful candidates, so that this branch of nursing is also flourishing. All the American training schools are formed after our English systems; but where we once led the van we will shortly fall to the rear, if we are not careful. The establishment of directories, provident funds, sick-cookery kitchens, and a thousand small conveniences and advantages we are trying to make known here through our pages, will shortly throw our training schools in the shade if we do not also all try to help forward the march of progress.

### Everybody's Opinion.

[*Correspondence on all subjects is invited, but correspondents must send name and address, and write on one side of the paper only.*]

### THE LIFE OF A PRIVATE NURSE.

### The Wearing of a Uniform.

In answer to "Islands" question, "What life is there so lonely as a private nurse's?" I should reply, "That of a governess." Not the highly-finished certificated woman taking advanced pupils, mingling in society as their companion, but the average instructress who takes "entire charge," and receives a remuneration of £25 to £30 per annum. Of course no hard and fast line can be drawn, but the chances are that where the governess spends her day in contact with the half-developed minds of children, the nurse may be on duty with some irritable patient, who nevertheless, in the quieter moments free from suffering, shows clear proofs of a refined or highly-gifted intellect and mind. Of course hospital work is brighter—more companions, more "cases," more professional competition and excitement; granted that in private nursing one continually goes over the same ground, that the days whereon you can only watch and wait come round very often, possibly monotonously; still every nurse can make an effort not to settle into a groove. In most houses you can obtain the daily papers; these alone, if read, prevent a nurse from remaining in ignorance of "what goes on outside her nursing world." In houses where the various inmates monopolise the day's *Standard* or *Times*, I take it for perusal the following day, choosing rather stale news than none at all; or if the paper disappears every morning with the head of the house, to that mysterious bourne, "The City," then I console myself with an hour's reading of Spanish, or any other foreign tongue in which I am far from being at home.

I cannot think the question of wearing a uniform can be objectionable to any sensible nurse. I often think of our matron's word: "When you grow ashamed of wearing your uniform you deserve to cease being a nurse." As regards "being months at a case with an old lady or gentleman," possibly far in the country, that is exactly my present position: instead of the dreary programme "Islands" sketches, I spend quite an enjoyable day. The "case," from being an apparently hopeless one, despaired of by doctor and physician, has gradually crept back to convalescence. The patient is able to be left for an hour or so in non-professional hands. I, the poor "lonely private nurse," may, for recreation, share in tending the flower garden, discuss the attractive contents of the kitchen ditto, or if I choose, practise, the piano being freely placed at my disposal; and often I am offered a spare seat in the carriage, and have a most enjoyable drive. True, the daily walk is alone, so far as biped companionship goes, but I supplement this—shall I say deficiency?—by inviting the two dogs out for exercise, and with the boisterous, grateful quadrupeds I cannot say I feel alone. This is not an isolated case, painted *couleur de rose*. I am not juvenile enough to see things in the glamour of novelty or of youth. I have had my troubles, but I thank God for the day I became a nurse, and think the life a noble one. I do not know if "Islands" is one of us, but if so, surely experience would teach that no nurse expects "people around her to lighten her responsibility." With the exception of the first private case, it is not overpowering: the nurse has been trained to it, it comes upon her gradually, first as a probationer, when she always had her seniors at hand ready to advise, gradually increasing with her knowledge and efficiency, but so gradually increasing as not to overpower. And, in conclusion, a word as to the conduct of a private nurse. She must at times ignore her professional dignity: it may be she has to stand and watch the amateur, and more or less clumsy efforts of some relative in the sick-room. She can, however, afford to do this; if she prove her training has not been for naught, the patient will soon prefer her services to those of any other. She must remember the roof she is under belongs to those who have a right to follow their individual ways, and show the deference an ordinary guest pays to his host; and I, for one, see no hardship in having to fall into the ways of the house.

Nurse Waller.

### A Mistaken Vocation.

Anent the article appearing in last week's issue of the "Mirror," surely it is hardly fair to take such a gloomy view of the matter. If uniform be such a great objection, we need not join an institution where its out-door use is imperative, and there are plenty of institutions where it is not so. In fact, we are not obliged to be nurses at all unless we choose. Why adopt a profession and then murmur at the requirements of it? Perhaps it is not the most lively path of life, but whatever we do we must expect to find some drawback. May I add that when a nurse (let her be in hospital or private work) finds her work irksome, and her life lonely, she should be convinced that she has mistaken her vocation. Much has been said as to the trials and tribulations of the private nurse, but little or nothing as to what her qualifications should be. Some of the best of hospital nurses are quite unsuitable for private work. I think that a private nurse should be a woman of large and varied experience—not, as is too often the case, a nurse of a few months' training—with a thorough knowledge of household management, and tact to cope with any emergency; and, above all, she should be able to prepare and cook any food necessary for her patient. If she is not required to do it, so much the better. I know we are often at cases where there is no need for us to do anything out of the sick-room; but, on the other hand, how often we are sent to a case to nurse the lady of the house, perhaps in only a very small establishment. What a relief it must be to her to find that, in addition to a competent nurse, she has someone who can take the management of her household affairs. Some of my readers may say that it is sufficient to have charge of the patient, but by being able to save your patient from domestic anxiety you help to complete your own special work of nursing. We cannot afford to stand on our dignity too much, or consider whether this is, or, that is not, the work of a nurse, because it is menial. "Whatsoever thy hand findeth to do, do it with all they might." As a private nurse, I have had to do almost anything and everything, from book-keeping etc., down to the washing of linen from a bad case of confluent small-pox. I can imagine how horrified you are, my readers, and no doubt would assert that there could be no possible necessity for me to do the latter, but I can prove that there was: the wife of my patient was a delicate woman; she did it once, but never having seen the like before, it made her sick and ill; I then considered it my duty to do it, as we could not hire anyone, owing to the nature of the disease. I don't wish anyone to think I prefer such work to my own, but to be the help to a family, which is looked for from a private nurse, you need to do many things which are quite apart from your work as an ordinary nurse. T. A.

### The Other Side.

I have read with much interest "The Life of a Private Nurse," by "Islands," in your issue of last week, and am inclined to think a more gloomy view of the matter has been taken than is necessary. I am a private nurse, and have been so sufficiently long for "the novelty" to have completely worn off. Consequently, I too may speak from experience.

Firstly. If a nurse is so "absorbed in her work" that she no longer shows the same real interest in her home as heretofore, so that her friends in consequence are disappointed, surely there is something radically wrong in the nurse.

How can she expect others to enter into her work, aims, and plans, if she, in her turn, does not show that ready sympathy and interest in theirs? From "Islands'" point of view the private nurse seems rather a selfish woman, possessed of but one solitary idea, and whose attention can only be aroused when her own work is the subject under discussion, and whose interest fails when other topics are introduced. Surely, the fault lies in the nurse, and not in the "life" she has entered upon. Let her cultivate to the utmost an intelligent interest in all that is passing around her, and with "a heart at leisure from itself," she will find her own life grow fuller and happier, because filled with the fulness of others. Why should she not look on the bright side—for there is a bright side to her life—and though often "tired," she will not feel "lonely," though sometimes she will have moments of depression and disappointment (for who has not?), yet the habit of cheerfulness carefully cultivated will stand her in good stead "till the clouds roll by." Hume, the historian, wrote: "To be happy, the person must be cheerful and gay, not gloomy and melancholy." A propensity to hope and joy is real riches, and not dependent upon time, place, or position.

Secondly. "Islands" makes a broad and mournful statement that private nurses form few lasting friendships. This is rather too broad to be covered; but admitting that such is the case so far as her patients are concerned, it would be well to seek for reasons other than lack of opportunities for meeting former patients and their relations. Whilst most of the great hospitals are nursed by well-educated women and ladies, provincial nursing institutions are mainly recruited from the ranks of respectable tradesmen's daughters, who, however intelligent and capable as nurses, are scarcely fitted intellectually and socially to form lasting friendships with their patients. But private nurses, from my personal experience, seem far from lonely or friendless. Some little time ago, when taking holiday duty for our matron, I was struck with the number of letters I forwarded to nurses. Many of these were from former patients; and though perhaps few of them may have belonged to the type of lasting friendships, yet to us they are like fragrant flowers on our life's journey.

Thirdly. "Islands" says, the nurse "seldom marries," as though this were a matter for either gloom or surprise, and the reason given is, because she lives in a "little world of her own," and apparently does not get the chance. Poor thing! what with being slighted by male patients, and no opportunities of meeting others, it must be a wonder to the reflecting mind, how it comes to pass women are still desirous of entering on such a vocation. To me the marvel is that there are exceptions to the rule. The fact is, women who have their own living to earn taste a delightful sense of freedom and independence in spite of hard work and anxiety, and it seems to me that the constant changes in a private nurse's life unfit her for the settled, prosaic duties of the married state, and probably she feels this. Be that as it may, few nurses having put their hand to this plough draw back; there is a fascination about their work which endures and increases to the end, and keeps them at a post which to outsiders may seem hard, but to the true nurse is full of interest, and in doing her duty she finds true happiness.

I should just like to say a word about the Home to which I belong. Our matron may be an exception, but if so, we are fortunate in having her. She does all in her power to make it home to us when off duty, and, when time and opportunity permit, we are allowed any recreation we desire within reason.

Handclasp.

---

A Dutch missionary in Africa bought a negro boy two years old, who was deaf and dumb, for six pounds of salt. On account of his infirmity, but for the interposition of this missionary, the child would have been put to death. He is now a pupil in the institution for deaf mutes at Maestricht.

### Appointments.

[It is requested that successful candidates will send a copy of their applications and testimonials, with date of election, to The Editor, The Lodge, Porchester Square, W.]

Salop Infirmary.—We are glad to hear that Miss Oldham, late of Cheltenham, has secured the appointment of Lady Superintendent at the Infirmary in Shrewsbury.

Worcester Infirmary.—Miss M. J. McLelland has been appointed matron of the above infirmary, out of fifty candidates. Miss McLelland was trained at the Glasgow Home for Nurses, where she spent a pleasant five years, and was very successful in pleasing the doctors for whom she worked. For two years Miss McLelland was at the West Malling Institute, and then at the County Asylum, Littlemore. This last place Miss McLelland left at the end of 1883, to undertake the more onerous duties of matron at the Bradford Infirmary. Some of Miss McLelland's testimonials from Scotch physicians are simply excellent, and we congratulate Worcester on its new matron. We hope Worcester has secured an equally agreeable house-surgeon, and that there is prospect of peace for the poor nurses.

### HOW TO SPEND A HALF-HOLIDAY.

It is not such an easy thing as one might fancy, to know how to spend a half-holiday during this wet weather; tennis, cricket, boating, all the usual sports, are stopped by the continual rain, and the little people who had looked forward to Saturday's emancipation from lessons, remain at home grumbling and disgusted. They have done German Reed's entertainment and the Moore and Burgess Minstrels earlier in the season, and now their Saturday afternoons are often passed in watching the rain. But there is one thing which many little people have not done, and which ought to be most interesting to them, and that is, to go to service at the Great Ormond Street Hospital for Sick Children. Every Saturday afternoon at a quarter to five there is a short bright service in the exquisite little chapel within the hospital, and the public (especially its smaller members), are warmly welcomed, and provided with seats and books. There are all the nurses in their pretty uniforms, and numerous little patients in scarlet dresses and quaint three-cornered caps. One wee mite tries to lift a Prayer-book, but the thin little hands are too weak even for this, and the book falls with a crash. The noise does not seem to annoy the congregation though, it only calls up pitying tears to the eyes of a lady visitor. The hymns are all suitable for children, and there is one special prayer for the poor suffering babes in the wards around. And then when the service is over the nurses pick up their small charges and carry them away, and surely the sight of a sick child cuddling down in the nurse's arms is one we should all see and remember. Should there come another wet Saturday afternoon we hope some of our readers will remember Great Ormond Street, and go early to the hospital to see the wards, and then stay for the beautiful little service. We are quite sure they will never accuse us of having tempted them to spend a dull afternoon.

### Vacancies.

The following vacancies are announced:—

**Matrons.**

Haverfordwest Infirmary.

West Cornwall Infirmary; £40.

St. Albans Hospital; £40.

**Lady Superintendent.**

Bradford Infirmary; £60.

**Nurses.**

Western Fever Hospital; £22.

St. Heliers Institution.

Victoria Home, Bournemouth.

Albert Institute Devonport; £25.

Female Hospital, Woolwich.

Nurses' Home, Truro.

Workhouse Infirmary Nursing Association.

Clapham, Brixton, and Surrey Association.

Ryde Institution.

Kingston Home, Hull.

Douglas Cottage Hospital; £25.

Hospital for Epilepsy and Paralysis £20.

Gravesend Hospital.

Arbroath Infirmary; £20.

Home for Trained Nurses, Frome.

Southport Trained Nurses' Home; several.

Kent Nursing Institution; several.

**Probationers.**

Isle of Man General Hospital.
Cottage Hospital, Wallasey, Cheshire.

**Wardmaid.**

Cottage Hospital, Wallasey, Cheshire.

